# Addiction: From Context-Induced Hedonia to Appetite, Based on Transition of Micro-behaviors in Morphine Abstinent Tree Shrews

**DOI:** 10.3389/fpsyg.2016.00816

**Published:** 2016-06-07

**Authors:** Ying Duan, Fang Shen, Tingting Gu, Nan Sui

**Affiliations:** ^1^Key Laboratory of Mental Health, Institute of Psychology, Chinese Academy of Sciences, BeijingChina; ^2^Institute of Psychology, University of Chinese Academy of Sciences, BeijingChina

**Keywords:** tree shrews, addiction, morphine, CPP, micro-behaviors, transition, palatable food

## Abstract

Drug addiction is viewed as a maladaptive memory induced by contextual cues even in the abstinent state. However, the variations of hedonia and appetite induced by the context during the abstinence have been neglected. To distinguish the representative behaviors between hedonia and appetite, micro-behaviors in abstinent animal such as psycho-activity and drug seeking behaviors were observed in morphine conditioned place preference (CPP). To confirm the different effects of reward between drug and natural reward, a palatable food CPP paradigm was compared in current work. After a 10-day training in CPP with morphine or food, the preference was tested on day 1, 14, 28, and the changes of micro-behaviors were analyzed further. Our data showed that tree shrews treated with morphine performed more jumps on day 1 and more visits to saline paired side on day 28, which indicated a featured behavioral transition from psycho-activity to seeking behavior during drug abstinence. Meanwhile, food-conditioned animals only displayed obvious seeking behaviors in the three tests. The results suggest that the variations of micro-behaviors could imply such a transition from hedonic response to appetitive behaviors during morphine abstinence, which provided a potential behavioral basis for further neural mechanism studies.

## Introduction

Drug addiction has been viewed as an aberrant learning of the association between drug and the context (Hyman, 2005). This association is so long-lasting that relapse could be triggered by the contextual stimuli even after long-term abstinence in human addicts (O’Brien, 1997; Daglish et al., 2001; Volkow et al., 2006). Thus, a better understanding of this association might be critical to the development of effective treatments against addiction (Crombag et al., 2008). However, the response induced by the context is controversial (Bolles, 1972; Carey and Gui, 1998; Bardo and Bevins, 2000; Huston et al., 2013). On one hand, the repeated use of drug in a particular environment generated the association between the context and the drug-induced affective effect such as hedonia (Carey et al., 2005). Thus the contextual stimuli might activate this hedonic effect directly by performing psycho-activity without drug. On the other hand, the association was also strengthened by the motivational properties of drug (O’Brien et al., 1993; Dalley et al., 2007) and the contextual stimuli might trigger appetite for drug or drug seeking in the drug-free state. It might be necessary to distinguish these two context-induced responses, because they depended on distinct anatomical and neurochemical substrates (Spiteri et al., 2000; Berridge et al., 2009; Smith et al., 2011).

The conditioned place preference (CPP) model was commonly used in preclinical studies to investigate the association between addictive drugs and the contextual stimuli. In CPP, the animal was alternatively confined in one chamber after drug injection or another chamber after saline injection. Following repeated conditionings, the animal was allowed free access to both chambers in a drug-free state, and the time stayed in drug-paired chamber was taken as the index of preference (Bardo et al., 1995). It was generally thought that the acquired place preference was driven by the motivation or appetite (Tzschentke, 2007), but morphine conditioned mice performed hedonic behaviors rather than appetitive behaviors in the preference test (Spiteri et al., 2000). The contrary results suggested it might be worthwhile to examine whether hedonia or appetite was actually induced by the context. Furthermore, the morphine CPP in rats could maintain at least 6 weeks (Mueller et al., 2002) and morphine CPP score in rats was even increased after a 2-week withdrawal (Smith and Aston-Jones, 2014). Meanwhile, cocaine self-administrated rats also performed the progressively increased seeking during abstinence (Lu et al., 2004). Moreover, clinical studies reported that cue-induced appetite was increased after an acute abstinence and the craving persisted even after a long period of abstinence (Gawin and Kleber, 1986). These results suggested that the abstinent state might increase appetitive behaviors, but the variation of hedonic behaviors was unknown. Therefore, we investigated whether hedonia or appetite was induced by context after morphine CPP training, and explored the variations of these responses during abstinence.

In addition to addictive drugs, many natural rewards including food could also form CPP (Tzschentke, 2007). A series of studies proved different brain mechanisms between the hedonic and appetitive effects of food (Berridge, 2009; Castro and Berridge, 2014) and mice displayed more seeking or appetitive behaviors after food conditioning (Spiteri et al., 2000). However, it was unclear whether the hedonic effect of food was induced by context after conditioning. Moreover, cue-induced appetite for sucrose in rats also increased during abstinence (Grimm et al., 2005), which implied the variation of context-induced responses might occur during food abstinence. Thus, we detected both hedonic and appetitive behaviors after palatable food training, then we examined the variations of these behaviors during abstinence. Both drug and food produced the reward effect through acting in the same reward circuits, but had different influences on the reward system (Pitchers et al., 2010). Therefore, the food CPP was used as a comparison for a better understanding about the responses induced by drug-related context.

In this study, we observed the micro-behaviors in tree shrews to investigate the variations of hedonia and appetite after morphine or palatable food conditioning. Tree shrews were suggested as potential candidates for addiction studies, phylogenetically close to primates (Fan et al., 2013). Based on the characteristic nature of tree shrews and previous behavioral studies, the psycho-activity characterized by number of vertical jumps was used as the index for hedonic behaviors, which was shown to increase significantly in tree shrews after morphine injection (Shen et al., 2014). Meanwhile, we counted the number of visits between different chambers as the index of seeking behaviors, which was widely used in rats to reflect the appetitive state (Mellgren and Olson, 1983; Mellgren and Elsmore, 1991). The observations were made on day 1, day 14, and day 28 following conditioning and compared with pre-test to investigate whether the context-induced responses were changed or not during abstinence.

## Materials and Methods

### Animals

A total of eight male tree shrews (*Tupaia belangeri chinensis*; 12–18 months old; 130–160 g) from the breeding colony at the Animal House Center of the Kunming Institute of Zoology were used in the experiments. Animals were individually housed in stainless cages (395 mm × 300 mm × 595 mm) attaching to the nest boxes (246 mm × 158 mm × 147 mm) under standard conditions (a 12-h light/dark cycle with light on from 08:00 to 20:00; Room temperature at 25°C). Food and water were provided *ad libitum*. All experiments were conducted during the light phase. Animals were handled through opaque bags once a day for a week before the experiment, thus they were habituated to the treatment of the experimenter.

The experiments were conducted according to the National Institute of Health, Guide for the Care and Use of Laboratory Animals, and the protocols were approved by the Research Ethics committee of Institute of Psychology, Chinese Academy of Sciences.

### Drugs

Morphine hydrochloride (Qinghai Pharmaceutical, China) was dissolved in sterile physiological saline (0.9% NaCl) to its final concentrations.

### Apparatus

The CPP apparatus was composed of three stainless steel chambers (395 mm × 300 mm × 595 mm) as chamber A, C, and B in a row. The chamber C was in the middle and could connect with the nest box. The walls of chamber C were removable, separating chamber A and B, respectively. The apparatus were mainly featured with color cues in different sides of chambers according to the well-developed visual system in tree shrews (Petry and Harosi, 1990). Chamber A had a yellow floor with yellow and white horizontal stripes on the walls. In contrast, chamber B had a blue floor with blue and white vertical stripes on the walls. Chamber C just made by stainless steel without any decoration. All chambers had cameras mounted on the top to record the animals’ behaviors.

### Procedure

The procedures were made, respectively, in the morphine- and food-conditioned group. The procedures of two groups both consisted of three phases: pre-test, conditioning and post-tests on day 1, 14, 28 after last conditioning sessions. The difference in two groups was conditioning phase. The morphine CPP procedure was based on our previous study with minor modifications (Shen et al., 2014) and the food CPP procedure was referred to rat study (Kanoski et al., 2011). The timeline of the experiment was showed on **Figure 3A**.

#### Pre-test

The removable walls of chamber C were opened with a 5 cm width gap, and the tree shrews were placed in the chamber C through their nest boxes. Chamber C was the start for exploring the apparatus. Animals moved freely in the three chambers for 60 min on three consecutive days for habitation and pre-test. When the tree shrew was placed into the apparatus, it might hide in the nest box and did not explore the area for a while because it was sensitive to the change of the environment. Therefore, data acquisition started when the tree shrew first went out from the nest box and lasted for 30 min. Time spent in each chamber, the numbers of visits to each chamber and the numbers of vertical jumps on the third day was recorded as pre-test data. The biased procedure was used in our study, and the disliked chamber for each animal was used as the reward-paired chamber during conditioning training.

#### Conditioning

After the pre-test, the animals were randomly divided into two groups (*n* = 4 per group) to form morphine or food CPP, respectively. The details were described as follows.

Morphine-conditioned tree shrews were injected with 5 mg/kg (intramuscular injection, IM) morphine and placed in their paired chamber on the first conditioning day. This dose was only used for the first morphine injection and for the following morphine injection the dose increased to 10 mg/kg. This design was based on our previous results which showed that one morphine injection of 5 mg/kg could make tree shrews adapt to the strong pharmacology effect of morphine and avoid the potential harm induced by the increased dose in recent sessions. Twenty-four hours after the morphine injection, the tree shrews were injected with saline (1 ml/kg, IM, the same volume as the morphine injection) and confined to the other chamber. On the subsequent conditioning days, each tree shrew trained for eight consecutive days with alternate injection of morphine (10 mg/kg, IM) and saline. The interval between injection and putting animals into the chamber was 3045 min and the conditioning time was 90 min. The time was based on the previous study to make sure that animals stayed in high locomotor level after morphine injection.

The food CPP was designed to compare with the morphine and the only difference between the procedures was the rewarding event. Since food deprivation might change the motivational state and locomotor activity of animals, the palatable food CPP in the normal feed state of tree shrews was used in our study. Our preliminary study found that apple was their favorite among three kinds of food (dry yellow mealworm, apple and food pellets). Therefore, during the conditioning training (Day -9 to 0), tree shrews had free access to a piece of apple which was placed in the middle of the reward-paired chamber for 30 min and were conditioned with nothing in another chamber for 30 min on alternate day.

#### Test

After conditioning, the tree shrews freely explored the apparatus with the walls of chamber C opened to test their preference (P1-test). The procedure was similar to the pre-test phase. Moreover, to explore the preference and context induced behaviors during abstinence, tests were taken every 14 days (P14-test, P28-test).

### Statistical Analysis

Conditioned place preference score [time in reward-paired chamber/(time in chamber A + time in chamber B)] was the index of preference. All data was shown as mean ± SEM. The statistical package SPSS 19.0 was used for data analysis. Paired *t*-test was performed to examine the establishment of CPP and behavioral changes between pre-test and P1-test. One-way ANOVA for repeated measures and LSD *post hoc* were performed to examine the persistence of the preference and the behavioral results within three abstinent time points in each group. The accepted level of statistical significance was *p* < 0.05.

## Results

### Establishment of CPP and the Variations of Micro-behaviors after Conditioning

**FIGURE 1:**
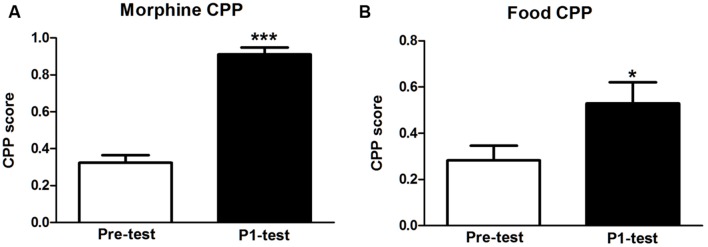
**Establishment of morphine and food CPP in tree shrews. (A)** Morphine conditioned tree shrews showed a significant preference for morphine-paired chamber. **(B)** Tree shrews in food group showed a significant preference for food paired chamber. Blank and solid columns represent data from pre- and P1-tests, respectively. Data were expressed as the means ± SEM, *n* = 4. *p < 0.05, ***p < 0.001.

After five alternating conditioning sessions, CPP score in morphine-rewarded tree shrews was increased significantly compared with pre-test (*t* = 15.560, *p* < 0.001). And CPP score in food group also displayed a significant increase compared with their Pre-test data (*t* = 3.215, *p* = 0.025). The results indicated that both morphine CPP and food CPP were established in tree shrews (**Figure 1**).

**FIGURE 2:**
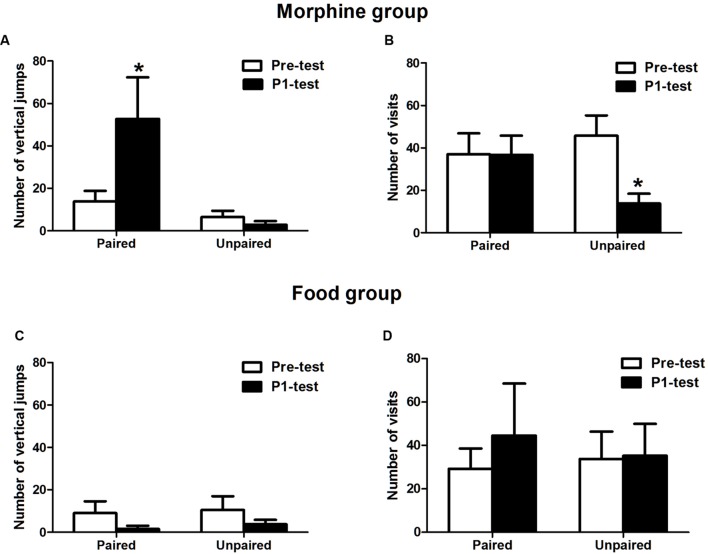
**Variations of psycho-activity and seeking behaviors on morphine and food CPP. (A)** The vertical jumps of the morphine conditioned groups increased in morphine-paired chamber. **(B)** The visits number of the morphine-conditioned tree shrews decreased in saline paired chamber. **(C)** The vertical jumps number of the food-conditioned tree shrews was stable in both chambers and decreased compared to Pre-test. **(D)** The visits number in food group maintained stable. Data were expressed as the means ± SEM, *n* = 4. *p < 0.05.

During the expression of CPP, the behavioral data was further analyzed to distinguish hedonia or appetite induced by the context (**Figure 2**). In the morphine-conditioned group, the number of vertical jumps significantly increased compared with pre-test in morphine-paired chamber (*t* = 2.665, *p* = 0.038) but not in saline-paired chamber (*t* = 1.071, *p* = 0.181), indicating that the high psycho-activity induced by conditioned context occurred in P1-test. Meanwhile, the number of visits decreased significantly in saline-paired chamber (*t* = 3.345, *p* = 0.019), but no significant difference in morphine-paired chamber (*t* = 0.051, *p* = 0.963), compared with pre-test. The decreased visits implied that not obvious seeking behaviors were induced by context. In the food group, the number of vertical jumps in both chambers was low and not changed (in food-paired chamber: *t* = 1.338, *p* = 0.136; in no food-paired chamber: *t* = 1.495, *p* = 0.116; compared with pre-test, respectively), suggesting no significant psycho-activity induced by the context. Meanwhile, the number of visits in both chambers was also stable compared with pre-test (in food paired chamber: *t* = 0.991, *p* = 0.197; in no food paired chamber: *t* = 0.214, *p* = 0.422). This result indicated that the food group expressed seeking behaviors, because the number of visits was not decreased even after habituation.

Above results showed that the morphine-conditioned group mainly performed hedonic behaviors, but the palatable food-conditioned group mainly performed appetitive behaviors on the expression of place preference.

### The Rewarding Value of Morphine or Food during Abstinence

**FIGURE 3:**
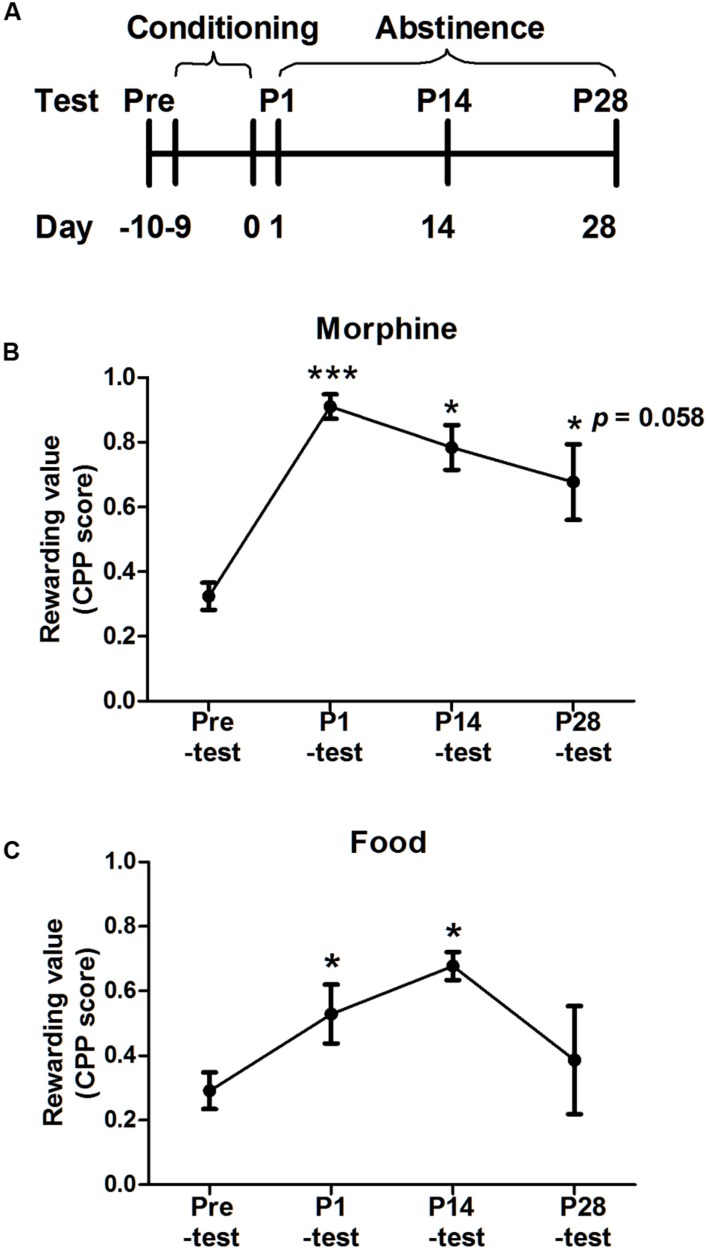
**Place preference induced by morphine or food during abstinence. (A)** Diagram outlining the behavioral procedures. After conditioning and P1-test, both groups were tested for CPP every 2 weeks during abstinence. **(B)** Morphine conditioned group maintained preference for 28 days. **(C)** Food conditioned group maintained for 14 days. Data were expressed as the means ± SEM, *n* = 4. *p < 0.05, ***p < 0.001, compared with their pre-test.

The CPP score was an index to reflect the rewarding value, which implied the strength of reward effects associated with the context. More importantly, both hedonia and appetite were induced by this reward effect. Thus, the place preference was examined every 14 days after conditioning (**Figure 3**). In the morphine-conditioned group, one-way ANOVA with repeated measurement revealed a main effect of test time [*F*_(3,12)_ = 13.040, *p* = 0.001], and LSD *post hoc* analysis showed significant differences in P1-test (*p* = 0.001), P14-test (*p* = 0.014) and a marginally significant difference in P28-test (*p* = 0.058), compared with pre-test. In the food-conditioned group, the main effect of test time was significant [*F*_(3,12)_ = 5.191, *p* = 0.024], *post hoc* analysis found the significant difference in P14-test (*p* = 0.005) but not P28-test (*p* = 0.519), compared with pre-test.

Above results implied the morphine-induced place preference could maintain for almost 28 days in tree shrews and the palatable food-induced place preference could last 14 days. Thus, the further micro-behavior was analyzed till on day 28.

### The Transition from Psycho-Activity to Seeking Behaviors during Morphine Abstinence

**FIGURE 4:**
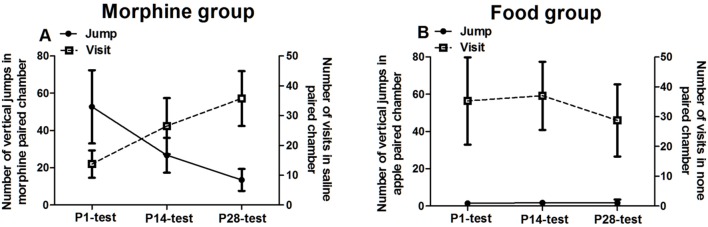
**Variations of psycho-activity and seeking behaviors during the abstinent state.** The left *y*-axis showed the number of vertical jumps in morphine/food paired chamber and the right *y*-axis showed the number of visits to saline/no food paired chamber. **(A)** In morphine conditioned tree shrews, the jumps number in morphine-paired chamber expressed decreasing trend, however, the visits number in saline paired chamber expressed significantly increasing trend during abstinence. **(B)** In food-conditioned tree shrews, the number of jumps visits showed no change during the abstinent state. Data were expressed as means ± SEM, *n* = 4.

Beyond the results in the Section “The Rewarding Value of Morphine or Food during Abstinence,” we recorded the micro-behaviors of tree shrews at 10 min intervals during four tests (Supplementary Figure S1). More detailed descriptions about this supplementary figure were in the discussion component. Based on these analyses on behaviors changing with time, we took the vertical jumps in reward-paired chamber as the index of psycho-activity, and took the number of visits in no reward-paired chamber as the index of seeking behavior. Both indexes in each group were displayed simultaneously (**Figure 4**).

In the morphine group, although there was no significant test time effect on the number of vertical jumps in morphine paired chamber [*F*_(2,9)_ = 5.156, *p* = 0.106], the number of jumps displayed a declining trend along with the abstinent time. It indicated that the psycho-activity was decreased during abstinence. Meanwhile, morphine-conditioned tree shrews displayed a time effect on the number of visits in saline paired chamber [*F*_(2,9)_ = 9.582, *p* = 0.014] and LSD *post hoc* showed significant difference between P1-test and P28-test (*p* = 0.040), and between P14-test and P28-test (*p* = 0.011). It suggested that the seeking behaviors in morphine groups continually increased during abstinence. In food-conditioned tree shrews, by contrast, the number of vertical jumps and visits was stable during the whole abstinence sessions, indicating that the responses were no changed during abstinence.

From above results, the increased seeking behaviors with decreased psycho-activity meant a transition from hedonia to appetite induced by context during morphine abstinence. However, food-conditioned group displayed no behavioral change during abstinence.

## Discussion

Our results indicated that morphine-conditioned tree shrews expressed more psycho-activity on short term-abstinence, but more seeking behaviors on long-term abstinence, implying a context-induced transition from hedonia to appetite. As a comparison, the food-conditioned group did not experience this transition during abstinence. There were still some questions to be discussed.

Both morphine and food CPP in tree shrews were established after five alternating sessions. However, the morphine-conditioned group showed higher CPP score than food group, in agreement with studies in rats (Duarte et al., 2003; Tzschentke, 2007). It implied greater magnitude of reinforcing effects in morphine. The reinforcing effects needed dopamine (DA) system participating in (Volkow et al., 2011) and preclinical studies showed that the higher and faster DA signals accorded with stronger reinforcing effects (Schultz, 2010). Morphine could activate DA system in a rapid access way, but food influenced the same circuits in two more indirect ways (Alonso-Alonso et al., 2015). Therefore, different reward effects might make morphine evoke higher reinforcement than palatable food. Furthermore, the analyses on micro-behaviors showed that morphine-rewarded animals displayed more psycho-activity in drug-paired chamber, which was also observed in rats after morphine or heroin treatment (Parker, 1992; Paolone et al., 2007). On the contrary, the food-rewarded animals mainly displayed seeking behaviors, in agreement with the study in mice which found the food-conditioned animals mainly expressed seeking behaviors after training (Spiteri et al., 2000). The various influences on DA system between drug and food might be the neural basis of these different responses.

To explore whether the hedonia and appetite were changed or not with the increasing duration of abstinence, the most important thing was to find the appropriate behavioral indexes to represent the hedonic and appetitive responses. We counted the number of jumps and visits in both chambers at 10 min intervals during four tests (Supplementary Figure S1). The number of vertical jumps in the morphine-conditioned group was increased significantly in the P1-test, compared with pre-test. Moreover, the number in the morphine-paired chamber emerged an increasing trend (Supplementary Figure S1B), and the trend was the same as that of psycho-activity after morphine injection (Shen et al., 2014). Therefore, the number of vertical jumps in reward-paired chamber was taken as the index of psycho-activity. Meanwhile, the number of visits in different chambers was recorded, which was a common item as seeking behaviors in rats. However, the meaning of the visits to the reward-paired chamber was misleading (Huston et al., 2013). Both hedonic effects and the appetite could lead the approach to this chamber. Moreover, based on our observation, the trend of variation on visits number was different during each test. During the P1-test the visits number of the morphine-conditioned group performed significant differences in 0–10 min (*t* = 4.621, *p* < 0.05) and 10–20 min (*t* = 5.62, *p* < 0.05), compared drug-paired with saline-paired chamber (Supplementary Figure S1B). But during the P14-test, there was the significant difference only in the second 10 min (*t* = 4.041, *p* < 0.05; Supplementary Figure S1C), and there was no difference during the P28-test (Supplementary Figure S1D). It indicated that the difference on the visits to the morphine- and saline-paired chamber became disappeared, and the number in saline-paired chamber made the main contribution. Tree shrews performed more and more visits to saline-paired chamber, although still had the preference for morphine-paired chamber. It was indicated that the seeking behaviors occurred. Therefore, the number of visits in no reward-paired could be the appropriate index as appetite. In addition, we also found an interesting phenomena that when the animals showed high number of vertical jumps, the number of visits to no reward-paired chamber was low, and when performs low jumps, the visits number was high. It suggested that two responses might compete against each other.

During abstinence, the morphine-conditioned group implied a transition from hedonia to appetite. One of the possible reason for this transition was that the negative affective state was elicited because of morphine absence (Mucha, 1987). To get rid of the negative effects, animals might produce more appetite for drug and thus performed more seeking behaviors (Ahmed and Koob, 2005). Morphine-conditioned tree shrews in our study performed decreasing psycho-activity during withdrawal, which implied that some negative effects might emerge. However, to get rid of the negative state, the place preference would be turned into aversion. But the preference was still performed on our study. The alternative explanation for the increased appetite might be from the motivational shift during abstinence (Berridge and Robinson, 1995). Addictive drugs caused a sensitization in the brain systems which would be the biological base of the motivation bias induced by drug (Berridge, 2007; Kalivas, 2009). The sensitization could be long-lasting and even aggravated during withdrawal (Lee et al., 2013; Loweth et al., 2014). When tree shrews re-exposed to the context, the contextual stimuli might trigger the sensitized system into higher level of activation, and more appetite for drug might be also triggered. So the behavioral transition might reflect the variation in neural system. Further, the development of drug addiction has been believed to result in maladaptive neurobiological responses induced by drug within the mesostriatal DA systems and corticostriatal glutamate (GLU) systems of the brain (Tzschentke, 2001; Everitt and Robbins, 2005; Mameli and Lüscher, 2011), and two systems participated in different responses. DA release in nucleus accumbens (NAc) was necessary for psychomotor sensitization (Volkow et al., 2001; Everitt and Wolf, 2002), and imaging studies suggested increased DA in the striatum was associated with the “high” in humans (Volkow et al., 2007), implying the DA system activation was involved in the high positive effect. However, GLU was necessary for drug seeking behaviors (Cornish and Kalivas, 2000) and played a critical role of cue-induced appetitive behavior after long-term withdrawal (Loweth et al., 2014). Previous studies have shown reduced phasic dopamine function in (NAc) during drug withdrawal (Acquas and Chiara, 1992; Diana et al., 1995; Ahmed and Koob, 2005), whereas emerging evidences suggested that GLU system played more important role in long term synaptic adaptation during abstinence (Cornish and Kalivas, 2000; Kalivas et al., 2009). These results suggested that the neural change underlying addiction from DA system to GLU system, which might be reflected by the behavioral transition from hedonia to appetite.

As a contrast, the food conditioned group had no transition during abstinence. The stable number of jumps and visits implied that the appetite for food was continuously induced by the context. Although the same behavioral phenomenon was observed on mice study (Spiteri et al., 2000), the meaning might be different. In Spitert’s study, the mice was on deprived state, and the appetitive behavior could be promoted by the food deprivation (Pyke, 1984). However, in our study the apple was as the palatable food to induce place preference and food was provided in their cages *ad libitum.* The high number of visits might suggest that the palatable food was as the goal conditioned with the context, which was related to the DA system (Carelli, 2002). When the food-conditioned group re-exposed to the context, the goal-seeking system was activated and animals performed seeking behaviors. Different from the morphine-conditioned group, the behaviors in food group was stable and had no transition. Drug and food both could activate the mesolimbic DA system, but the function of activations and the circuit which processed the reward effects were largely distinct (Carelli et al., 2000; Pitchers et al., 2010; Cameron et al., 2014). Moreover, although drug and natural rewards could both induce adaptive synaptic adaptation, the changes of GLU receptors were in opposite direction during withdrawal (Cameron et al., 2014). These different neurobiological mechanisms might explain the variable behaviors between drug and food, and the specific effects induced by drug might be the molecular target for this transition.

The main finding in our study was that the morphine abstinent tree shrew displayed a behavior transition from psycho-activity to seeking behaviors, implying that context induced hedonia after short-term abstinence but appetite after long-term abstinence. It might remind the significance to take the abstinence time as an important factor to make more effective target for treatment on relapse.

## Author Contributions

YD and FS designed the experiments, preformed the experiments, analyzed data and wrote paper; TG participated in the writing of the article; NS designed the experiments and had primary responsibility for final content.

## Conflict of Interest Statement

The authors declare that the research was conducted in the absence of any commercial or financial relationships that could be construed as a potential conflict of interest.
