# Supplementary figures and images for: Addiction: From Context-Induced Hedonia to Appetite, Based on Transition of Micro-behaviors in Morphine Abstinent Tree Shrews
Source: Front Psychol. 2016 Jun 7;7:816. doi: 10.3389/fpsyg.2016.00816 (PMC4894903; doi:10.3389/fpsyg.2016.00816)

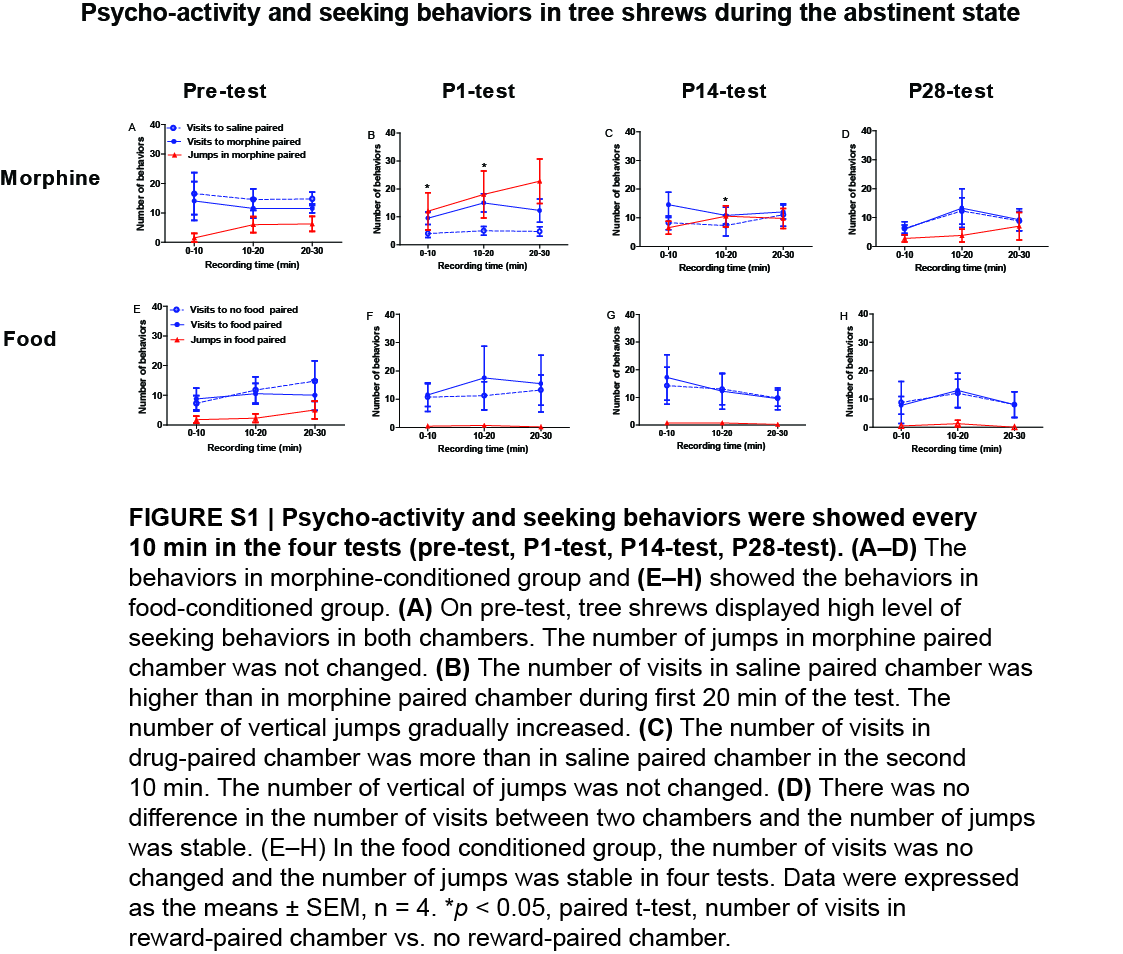

Supplement: Supplementary file 1 [file Image_1.TIF]
